# Adrenergic signaling controls early transcriptional programs during CD8^+^ T cell responses to viral infection

**DOI:** 10.1371/journal.pone.0272017

**Published:** 2022-08-09

**Authors:** Leonardo D. Estrada, Didem Ağaç Çobanoğlu, Aaron Wise, Robert W. Maples, Murat Can Çobanoğlu, J. David Farrar

**Affiliations:** 1 Department of Immunology, UT Southwestern Medical Center, Dallas, TX, United States of America; 2 Encodia Inc., San Diego, CA, United States of America; 3 Department of Bioinformatics, UT Southwestern Medical Center, Dallas, TX, United States of America; University of Iowa, UNITED STATES

## Abstract

Norepinephrine is a key sympathetic neurotransmitter, which acts to suppress CD8 ^+^ T cell cytokine secretion and lytic activity by signaling through the β2-adrenergic receptor (ADRB2). Although ADRB2 signaling is considered generally immunosuppressive, its role in regulating the differentiation of effector T cells in response to infection has not been investigated. Using an adoptive transfer approach, we compared the expansion and differentiation of wild type (WT) to *Adrb2*^*-/-*^ CD8 ^+^ T cells throughout the primary response to vesicular stomatitis virus (VSV) infection *in vivo*. We measured the dynamic changes in transcriptome profiles of antigen-specific CD8 ^+^ T cells as they responded to VSV. Within the first 7 days of infection, WT cells out-paced the expansion of *Adrb2*^*-/-*^ cells, which correlated with reduced expression of IL-2 and the IL-2Rα in the absence of ADRB2. RNASeq analysis identified over 300 differentially expressed genes that were both temporally regulated following infection and selectively regulated in WT vs *Adrb2*^*-/-*^ cells. These genes contributed to major transcriptional pathways including cytokine receptor activation, signaling in cancer, immune deficiency, and neurotransmitter pathways. By parsing genes within groups that were either induced or repressed over time in response to infection, we identified three main branches of genes that were differentially regulated by the ADRB2. These gene sets were predicted to be regulated by specific transcription factors involved in effector T cell development, such as *Tbx21* and *Eomes*. Collectively, these data demonstrate a significant role for ADRB2 signaling in regulating key transcriptional pathways during CD8 ^+^ T cells responses to infection that may dramatically impact their functional capabilities and downstream memory cell development.

## Introduction

Cytolytic CD8^+^ T cells play a critical role in immune responses to pathogens and can be harnessed to target cancer. Their activation and development into effector cells are guided by a variety of signals that include antigen recognition, co-stimulatory receptor activation, and soluble factors such as cytokines. Upon antigen recognition, CD8^+^ T cells rapidly divide and acquire critical effector functions including cytokine secretion and lytic activity that are essential for pathogen clearance. Once the infection resolves, most cells die through attrition leaving a small pool of diverse memory cells with the capacity for rapid expansion and effector function in the face of a secondary infection. Many cells of the immune system, including CD8^+^ T cells, express various neurotransmitter receptors [1,2], yet the role of neural signals in T cell function remains largely unexplored. As secondary lymphoid tissues are heavily innervated by post-ganglionic sympathetic neurons that secrete norepinephrine (NE), the sympathetic nervous system would be expected to play a pivotal role in immune regulation [2–4].

In previous studies, depletion of endogenous NE through chemical sympathectomy significantly enhanced the innate cytokine storm leading to exacerbated pathology during influenza infection of mice [5]. This hyper-inflammation was accompanied by significantly increased IFN-γ-producing CD8^+^ T cells during the primary infection phase, indicating an important role for NE in limiting the magnitude of both innate and adaptive T cell responses to viral infections. More recent studies have demonstrated an intrinsic role for NE signaling to suppress cytokine secretion and lytic activity in both mouse and human CD8^+^ T cells [6–8]. NE acted specifically and exclusively through the ADRB2 to modulate acute effector function. Thus, ADRB2 signaling plays a distinct role in limiting the magnitude of T cell-mediated primary responses.

Initial T cell activation pathways, such as antigen recognition and cytokine signaling, are critical to antiviral responses. We wished to understand how ADRB2 signaling impacted these early primary transcriptional responses of T cells to an *in vivo* virus infection. In this study, we assessed and compared detailed transcriptome changes of wild type (WT) and *Adrb2*-deficient (*Adrb2*^*-/-*^) CD8^+^ T cells throughout their responses to a viral infection and utilized novel bioinformatic tools to define regulatory elements controlled by adrenergic signaling. We found that intrinsic ADRB2 signaling in CD8^+^ T cells controls early transcriptional programs at all timepoints through the first 12 days of their response to Vesicular Stomatitis Virus (VSV) infection. Many of these dysregulated pathways belonged to known regulators of T cell function and development including cytokine signaling and response to pathogens. This study highlights a critical role for ADRB2 signaling in regulating dynamic transcriptome expression throughout CD8^+^ T cell antiviral responses.

## Results

**Fig 1:**
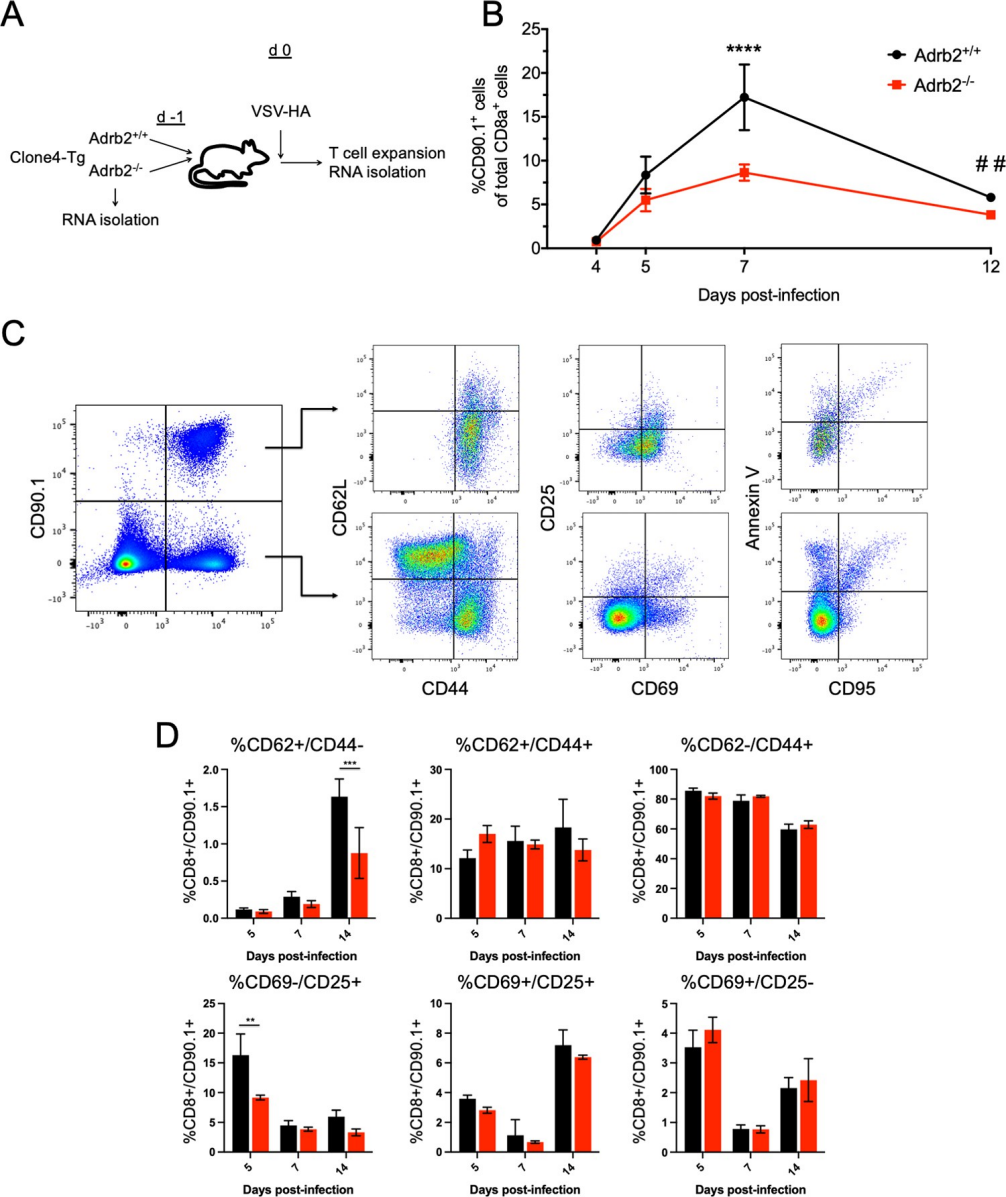
Differential expansion of Adrb2-/- cells in response to virus infection. (**A**) WT (CD90.1/1) and *Adrb2*^-/-^ (CD90.1/2) C4-Tg T cells were purified from donor animals and co-transferred (1:1, 1000 cells each) to BALB/cJ (CD90.2/2) recipients (n = 3 recipient animals/time point). On d1, recipient animals were infected with 1 x 10^6^ pfu VSV-HA. (**B**) Separate cohorts of recipient animals were sacrificed at the indicated days post-infection. Expansion of transferred cells in pooled spleen and lymph node was quantified by flow cytometry by measuring the percentage of WT and *Adrb2*^-/-^ cells within the total proportion of congenic CD90.1^+^ cells (**** p < 0.0001 and ## p <0.01 by two-way ANOVA). This experiment was performed 4 separate times with similar results. (**C**) WT and *Adrb2*^*-/-*^ CD8+ T cells were separately transferred to CD90.1 Balb/c recipients (3 recipient animals/genotype/time point) and infected with VSV-HA as above. Splenocytes assessed for cell surface receptor expression by flow cytometry. Cells were stained with antibodies to CD8, CD90.1, CD25, CD44, CD62L, CD69, with the addition of Annexin V to identify apoptotic cells. Expression of these markers were compared between the endogenous CD90.1-/CD8+ cells (lower panels) and the CD90.1+/CD8+ transferred cells (upper panels. (**D**) Percentage of specific CD90.1+/CD8+ populations assessed in C was quantified and compared between WT and *Adrb2*^*-/-*^ cells at each time point after infection. Significant differences were determined by 2-way ANOVA (5 separate similar experiments were performed).

To gain a better understanding of how ADRB2 signaling modulates peripheral CD8^+^ T cell development, we compared the expansion and gene expression changes that occurred in response to a virus challenge between WT and *Adrb2*^-/-^ CD8^+^ T cells. We utilized the clone4 T cell receptor transgenic (C4-Tg) model, which is specific for the influenza hemagglutinin antigen (HA) presented by H-2K^d^ on the BALB/c background [9]. We measured antigen-specific T cell responses to a recombinant VSV expressing HA protein from influenza (VSV-HA) [10]. In order to distinguish the responses, congenic WT (CD90.1/1) and *Adrb2*^-/-^ (CD90.1/2) C4-Tg T cells were adoptively co-transferred into BALB/c recipients (CD90.2/2), which then were infected with VSV-HA (Fig 1A). Primary expansion was monitored in spleen and lymph nodes by staining for both CD90.1 and CD90.2 congenic markers that distinguished the transferred from the endogenous pool of CD8^+^ T cells as well as WT from *Adrb2*^-/-^ cells (Fig 1B). WT and *Adrb2*^-/-^ cells expanded equally to day 5 following infection. However, we observed a significant reduction in *Adrb2*^-/-^ CD8^+^ T cells at days 7 and 12 post-infection compared to WT cells. We monitored a variety of cell surface markers correlated with the expansion of effector cells throughout the time course of infection. As shown in Fig 1C, the majority of antigen-specific CD90.1+ cells transitioned uniformly into effector cells expressing low levels of CD62L with high levels of CD44. This contrasted with the endogenous pool of CD90.1- CD8+ cells in which, as expected, only a subset of responding cells attained this effector phenotype. Further we observed no significant differences between WT and Adrb2-ko in the proportion of CD44+/CD62L+ or CD44+/CD62L- cells at any timepoint of virus infection (Fig 1D). In contrast, there was a significant reduction in the percentage of *Adrb*2^-/-^ CD62L+/CD44- (central memory-like) cells at d14 post-infection. This reduction in these central memory-like cells at d14 was preceded by a reduced proportion of CD69-/CD25+ cells at d5 post-infection. Regardless of whether WT and *Adrb2*^-/-^ cells were co-transferred to the same host or if they were separately transferred to different hosts, the results were consistent between the two experimental approaches.

**Fig 2:**
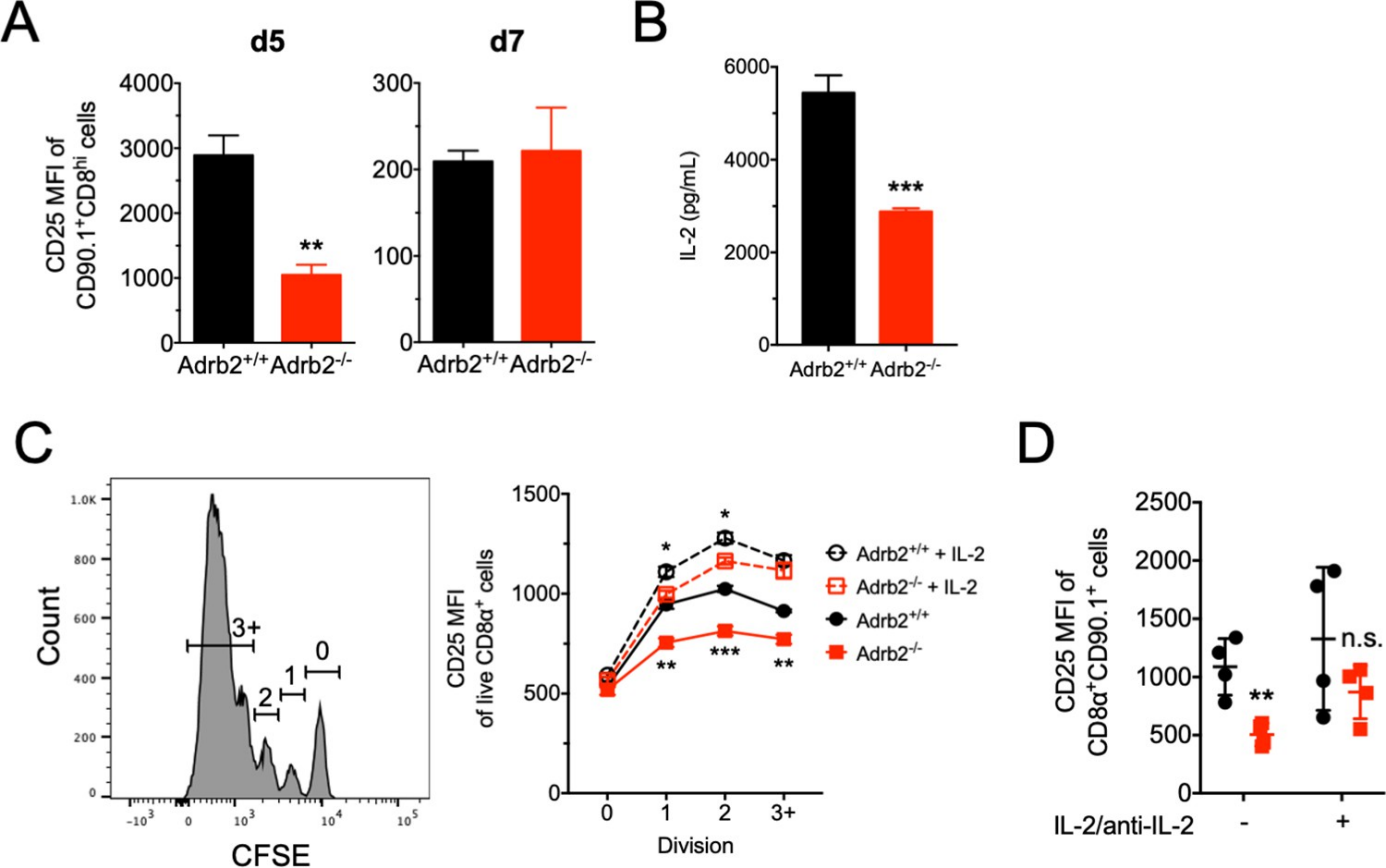
The *Adrb2* controls expression of IL-2 and the IL-2Rα. (**A**) WT and *Adrb2*^*-/-*^ cells were co-transferred to WT Balb/c recipients (n = 3 animals/time point) followed by infection with 1 x 10^6^ pfu VSV-OVA. WT and *Adrb2*^-/-^ cells were distinguished based on expression of CD90.1 and assessed for relative expression of the high affinity IL-2Rα (CD25) at days 5 and 7 after infection. (**B**) Spleen cell cultures from WT and *Adrb2*^*-/-*^ CL-4 TCR Tg animals were cultured with HA peptide for 24 hrs. IL-2 was measure by ELISA from the culture supernatants. Statistical significance was determined by Student’s t test. (**C**) WT and *Adrb2*^*-/-*^ CL-4 TCR Tg spleen cells were labeled with CFSE and activated for 72 hrs with HA peptide in the absence or presence of IL-2 (50 U/ml). Cellular divisions were gated based on dilution of CFSE (left panel), and relative expression of CD25 in each division was measured in live/CD8+ cells by FACS (right panel). Statistical significance was determined by two-way ANOVA, and differences between genotypes and treatment are indicated by each symbol at each time point. (D) WT and *Adrb2*^-/-^ CD8+ T cells were transferred to WT Balb/c recipients followed by VSV-HA infection, as described in (A). Cohorts of animals (n = 3 animals/group) were treated with either IgG2A/rmIL-2 (-) or anti-IL-2/rmIL-2 (+) conjugates on days 1, 2, and 3 post-infection. CD25 expression was measured on CD8+/CD90.1+ cells on d5 post-infection. Statistical significance was determined by two-way ANOVA. (All data are SEM, and * p≤0.05; ** p≤0.01; *** p≤0.001).

At the early time point of infection, the reduced percentage of CD25+ cells correlated with suppressed overall expression of the high-affinity IL-2Rα (CD25) on the entire population (Fig 2A) Further, we found that *in vitro*-stimulated cells from Adrb2-ko mice secreted significantly less IL-2 than WT cells. As IL-2 can act in an autocrine fashion to drive CD25 expression, we tested the ability of IL-2 to restore CD25 expression in *Adrb*2^-/-^ cells. Although we previously found no marked differences in either proliferation or apoptosis in response to antigen stimulation [11], we found that *Adrb2*^*-/-*^ cells displayed reduced induction of CD25 as a function of TCR-stimulated cell division *in vitro*, and supplementation of cultures with IL-2 restored CD25 expression in *Adrb2*^-/-^ cells (Fig 2C). To test the role of IL-2 signaling in vivo, adoptive transfer of WT and Adrb2-ko cells was performed in separate recipients, which were then infected with VSV-HA as described above. Cohorts of animals were then treated with either control IgG2a/IL-2 or with anti-IL-2/IL-2 conjugates on days 1, 2, and 3 of infection. We found a significant reduction of CD25 expression in *Adrb2*^-/-^ cells, which was restored when animals were treated with the IL-2/anti-IL-2 conjugate (Fig 2D). While IL-2 signaling restored CD25 expression, we found no significant difference in their overall expansion, suggesting that the ADRB2 may play a role in regulating other downstream pathways in parallel with IL-2 signaling.

**Fig 3:**
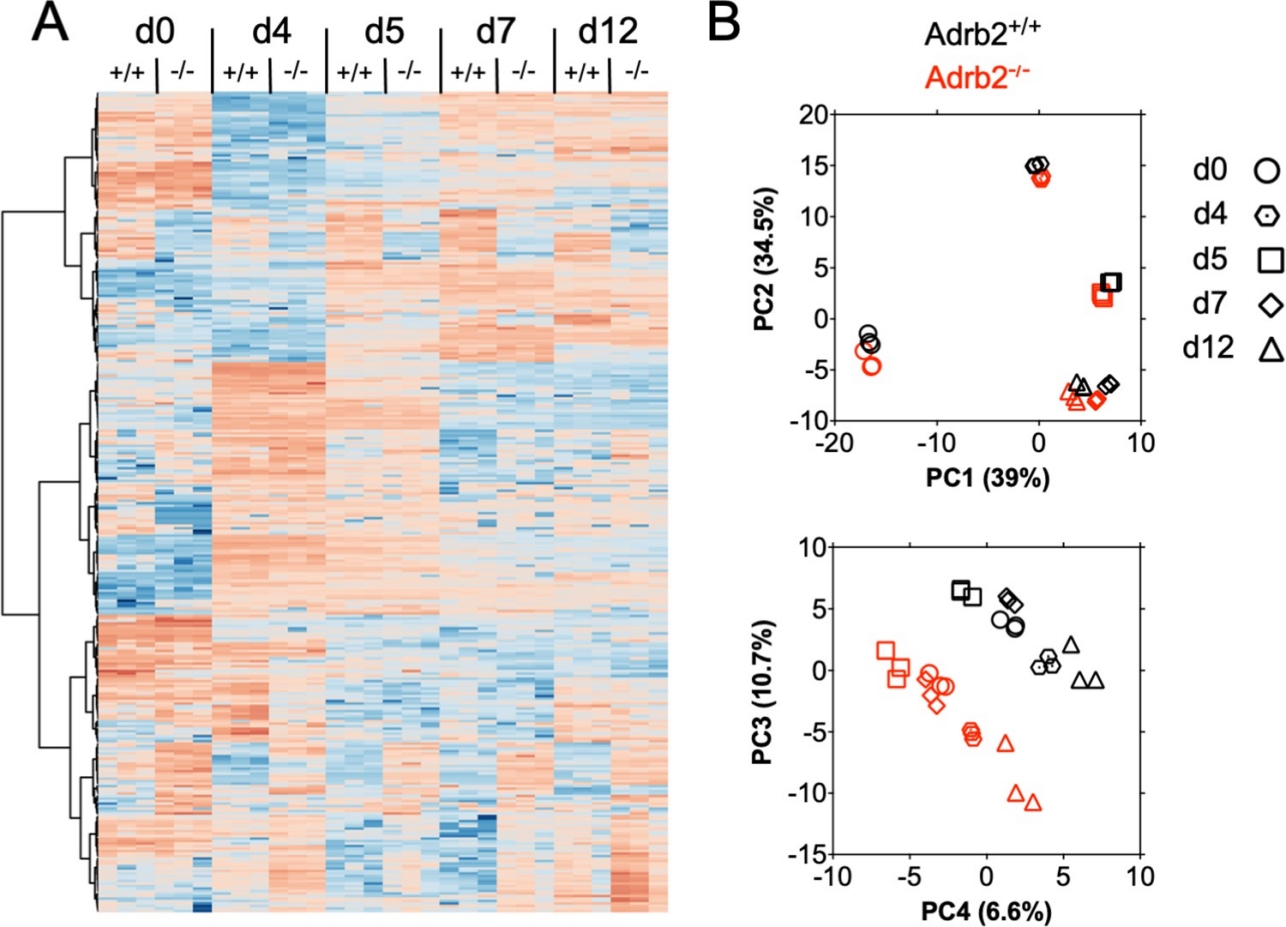
The ADRB2 regulates differential gene expression in CD8+ T cells during anti-viral responses in vivo. WT (CD90.1/1+) and *Adrb2*^-/-^ (CD90.1/2) CD8+ T cells were purified from recipient animals described in Fig 1A. RNA was isolated for RNASeq analysis from purified cells on day 0 and from infected hosts on days 4, 5, 7, and 12 post-infection. (**A**) EdgeR analysis identified all genes differentially expressed at any timepoint between WT and *Adrb2*^-/-^ cells. Gene expression values were used to perform 1-way hierarchical clustering, and data are displayed as a heat map. (**D**) Principal component analysis of differentially expressed genes between WT and *Adrb2*^-/-^ cells displayed as a function of each timepoint post-infection (PC1 vs PC2, top panel; PC3 vs PC4, bottom panel).

**Fig 4:**
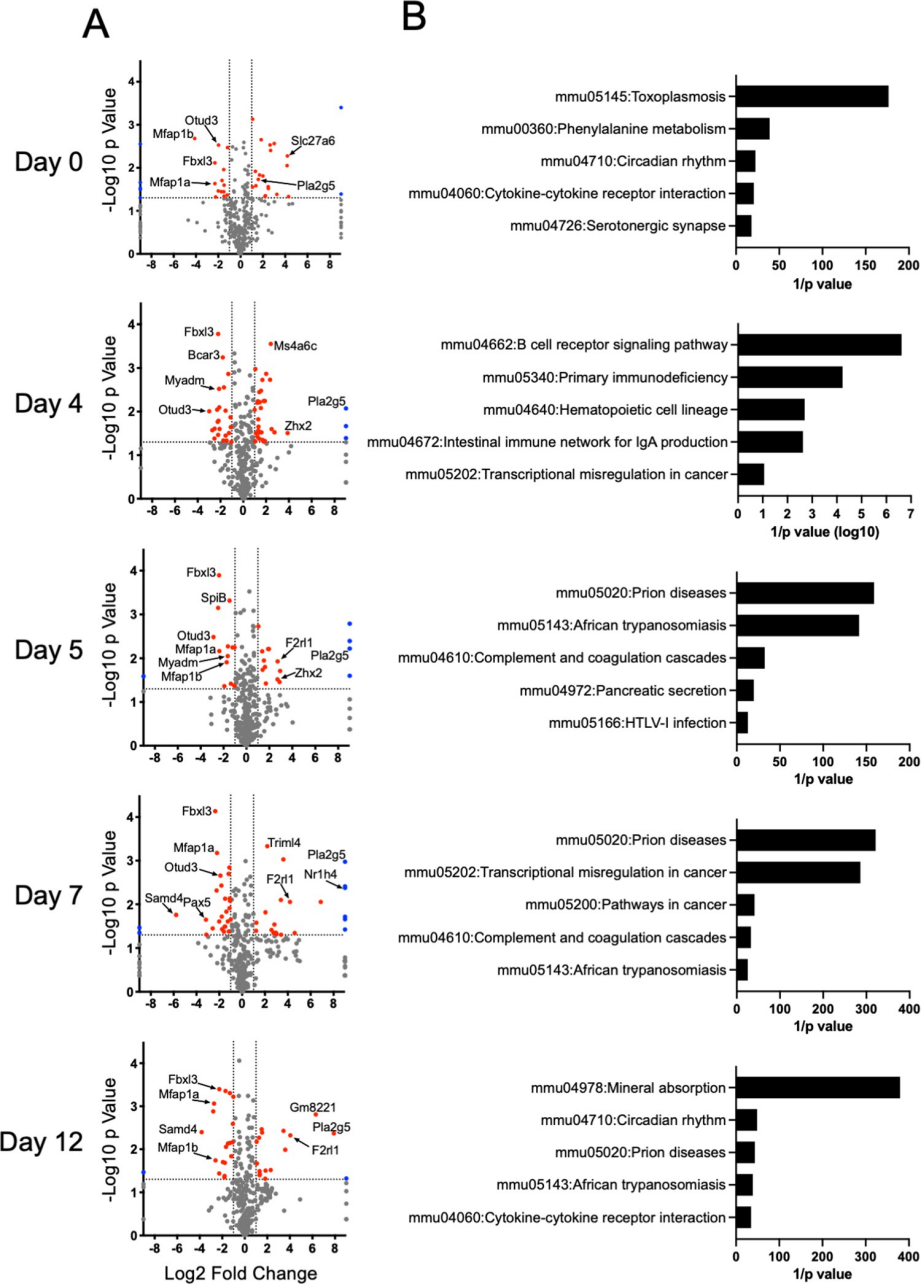
The ADRB2 selectively regulates transcriptional pathways involved in diverse immune functions. (**A**) Differentially expressed genes between WT and *Adrb2*^-/-^ cells at each time point of infection are displayed in volcano plots, and select genes contributing to major KEGG pathways are denoted within the plots. (**B**) KEGG pathway analysis was performed with the differentially expressed genes at each time point. The top five pathways are displayed. All pathways were found to be significantly enriched at p≤0.05.

Early T cell priming events regulate long-range transcriptional programs that lead to both effector and memory cell development [12,13]. We measured gene expression changes in FACS-purified WT and *Adrb2*^-/-^ cells from VSV-HA-infected animals at each time point shown in Fig 1B by RNASeq analysis. Over 6000 genes were collectively regulated in response to infection in both cell types. EdgeR analysis [14] identified over 320 genes that were differentially expressed between WT and *Adrb2*^-/-^ cells at any of the time points, including day 0 (S1 Table). A one-way hierarchical cluster of these genes demonstrated a temporal change in gene expression, which for some clusters of genes differed at all timepoints (Fig 3A). The temporal dynamics of gene expression changes were highlighted by principal component (PC) analysis of this gene set, as genes within the first two PCs differed significantly by time, but not by genotype (Fig 3B, upper panel). These components were driven by the expression of genes involved in the primary effector response such as cytokines, chemokines, and cytokine receptors. However, PCs 3 and 4 displayed a marked division over time between WT and *Adrb2*^-/-^ cells, which were comprised of genes involved in a variety of cellular processes including transcription, signal transduction, and cellular differentiation (Fig 3B, lower panel). We further segregated these temporal gene sets based on their up or down-regulation at each time point relative to WT cells, and select genes are annotated within the volcano plots in Fig 4A. Genes that were significantly differentially expressed, either positively or negatively, between WT and *Adrb2*^-/-^ cells were then assessed for their contribution to specific KEGG pathways (Fig 4B). We found that unique pathways were engaged by ADRB2 signaling at incremental times through their progression to effector cells. Among these pathways, we found that select pathways were dysregulated at multiple times throughout the early phase of infection, such as cytokine receptor interaction, transcriptional misregulation in cancer, and circadian rhythms. The genes driving these varied pathways are listed in S2 Table and included *Stat1*, *Il2ra*, *Il10ra*, *Per2*, *Fbxl3*, and *Mef2c*. Although the sorted populations of cells were determined to be >95% pure in post sorting analyses, we observed low-level expression of some B cell-associated mRNAs including *Cd19* and *Btk*, which were differentially expressed in *Adrb2*^*-/-*^ cells. It is possible that either there is low expression of these mRNA in CD8+ T cells, or there was low-level contamination with B cells in these samples. Overall, these data suggest a statistically significant role for the ADRB2 in regulating the temporal expression of genes during the early stages of T cell priming.

**Fig 5:**
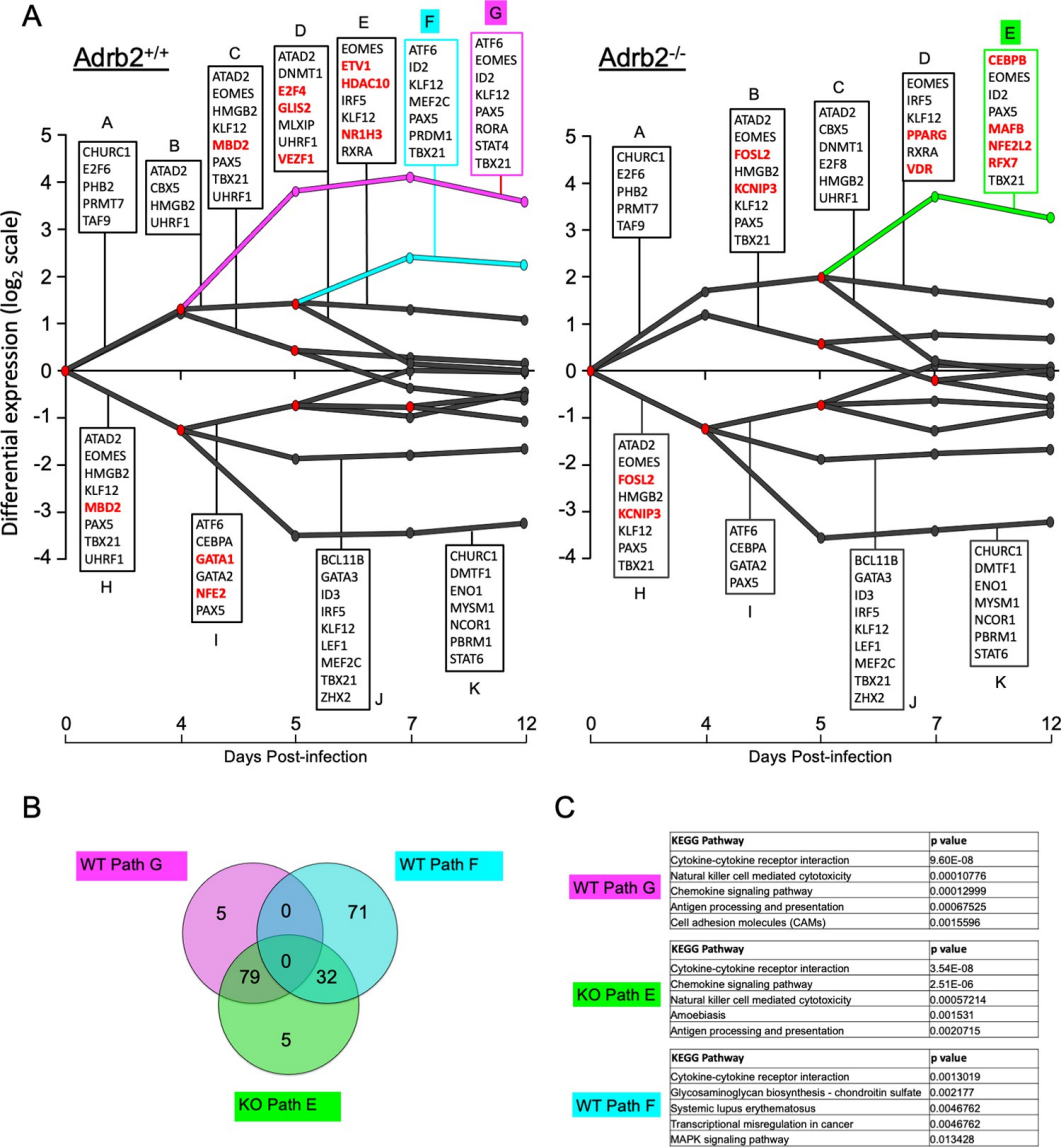
ADRB2 signaling coordinates temporal waves of gene expression. (**A**) SMARTS analysis at each timepoint compared to the day 0 pre-transfer condition. Each path corresponds to clusters of genes sharing common magnitude, direction of expression and regulatory factors. Red nodes in the model represent splits in expression between groups of genes. TRs predicted to regulate each split path are listed above and below their corresponding paths, and TRs in red were predicted by SMARTS to be involved in differential gene regulation between WT and *Adrb2*^-/-^ cells. WT paths F (cyan) and G (magenta), and *Adrb2*^-/-^ path E (green) contained genes and regulators which were significantly altered between the two models; they were subjected to further analyses (in B and C). (**B**) Venn analysis of constituent genes within WT paths F and G and *Adrb2*^-/-^ path E. Values represent the numbers of genes within each unique or shared segment. (**C**) Integrated KEGG pathway analysis of constituent genes of WT paths F and G and *Adrb2*^-/-^ path E. The top 5 significantly enriched pathways from each path are listed.

As cells divide and develop over time, their gene expression patterns become highly variegated. These fluctuations in gene expression can be modeled in terms of the regulatory dynamics that cause them. We utilized SMARTS to model, compare, and visualize the regulatory dynamics following infection for WT and *Adrb*2^-/-^ cells (Fig 5A) [15]. Using SMARTS, we constructed regulatory models for each condition using CD8^+^-specific transcription regulator (TR)-gene interaction data derived from Best et al. [16]. Genes were parsed into paths based on the similarity they share in both the direction and magnitude of expression at each time point, as well as the TRs which are known to regulate them. A full list of genes for each path is provided in S3 and S4 Tables. Each SMARTS model represents sets of genes following a similar regulatory program as paths; split nodes represent regulatory events that cause groups of genes to diverge in expression. Select TRs predicted to regulate genes within each path are listed in box diagrams for each path in Fig 3A, and a complete list is provided in S5 and S6 Tables. As expected, genes within both positively and negatively regulated paths shared regulation by select transcription factors known to be involved in CD8^+^ T cell function and memory development including *Tbx21*, *Eomes*, *Irf5*, *Rxra*, *Prdm1*, *Id2*, and *Stat4*. Gene regulation by these TRs was predicted by SMARTS to be shared between WT and *Adrb*2^-/-^ cells. SMARTS was also used to identify TRs that follow distinct regulatory programs in WT and *Adrb*2^-/-^ cells (S7 Table). TRs found to be involved in differential gene regulation within select paths are highlighted in red in Fig 3A. For example, *Mbd2* was predicted to selectively regulate genes in WT paths C and H, while *Mafb* was predicted to regulate genes within path E in *Adrb*2^-/-^ cells but not WT cells.

Overall, the pattern of gene expression changes that occurred over time were similar between WT and *Adrb*2^-/-^ cells. However, we identified a unique path consisting of genes that were more highly induced on day 4 post-infection in WT cells that were not regulated at that timepoint in *Adrb*2^-/-^ cells (Fig 5A, *Adrb2*^+/+^ path G (magenta)). By comparing the constituent genes of paths F (cyan) and G (magenta) in WT cells with path E (green) in *Adrb*2^-/-^ cells, the majority of genes in WT path G were included in the split path E in *Adrb*2^-/-^ cells (Fig 5B, S8 Table). Further, the genes in WT path G and *Adrb*2^-/-^ path E mapped to many of the top KEGG pathways involved in T cell effector function (Fig 5C). Despite their similar directional change, only 1/3 of genes within WT path F were shared with *Adrb*2^-/-^ path E, which was underscored by the unique KEGG pathways involved in WT path F. These data suggest that the effector response genes induced at day 4 post-infection in WT cells were either temporally delayed or absent in their induction in *Adrb*2^-/-^ cells.

**Fig 6:**
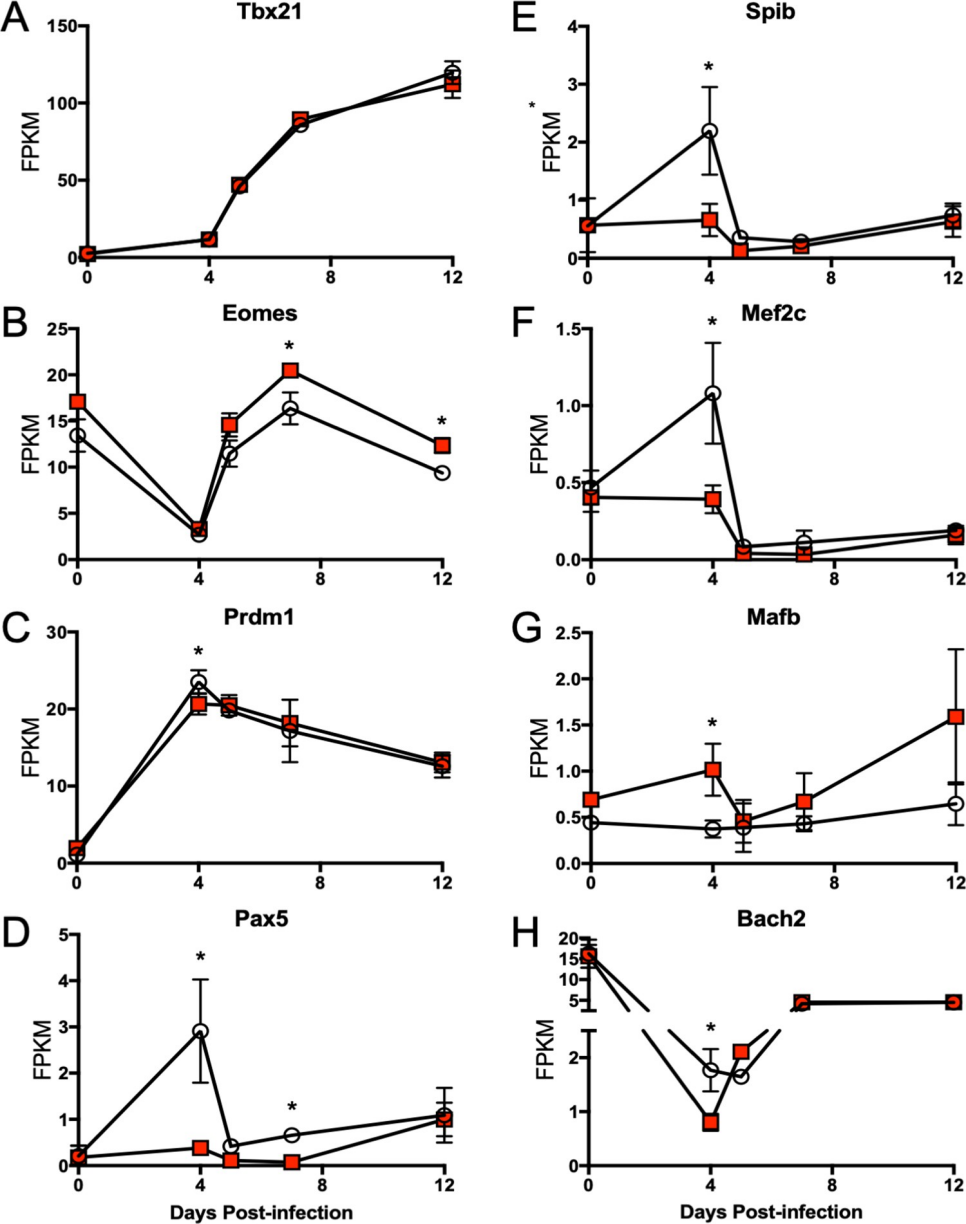
Select transcription factor expression regulated by the ADRB2. Gene expression data are derived from the RNASeq analysis described in Fig 3. Normalized mean (+/- SD) FPKM values for specific transcription factors are displayed for WT (open circles) and *Adrb2*^*-/-*^ (red squares) CD8^+^ T cells isolated at the indicated time points of infection. (A) *Tbx21*, (B) *Eomes*, (C) *Prdm1*, (D) *Pax5*, (E) *Spib*, (F) *Mef2c*, (G) *Mafb*, (H) *Bach2*.

Common to both paths in WT and *Adrb*2^-/-^ cells, *Tbx21* and *Eomes* were predicted by SMARTS to regulate genes known to be involved in the transition from effector to memory cells, yet *Tbx21* itself was not differentially expressed at any time point between the two models (Fig 6A). However, *Eomes* was more highly expressed in *Adrb*2^-/-^ than WT cells on days 7 and 12 post-infection (Fig 6B), which may impact the effector to memory transition based on previous studies. Within the gene sets of WT path G and *Adrb*2^-/-^ path E, we identified several TRs that were differentially expressed at day 4 post-infection, the time point at which the major split path occurred in WT but not *Adrb*2^-/-^ cells. These factors included *Prdm1*, *Pax5*, *Spib*, *Mef2c*, *Mafb*, and *Bach2* (Fig 6C–6H). Of these factors, *Pax5* and *Spib* are predominantly expressed in B cells and silenced in T cells [17,18], yet their transient induction in WT CD8^+^ T cells during infection may indicate a previously unanticipated role for them in T cell function. However, *Prdm1*, *Mef2c*, and *Bach2* have been shown to regulate various aspects of T cell function [16,19,20], specifically the effector to memory cell transition. Of note, *Prdm1* was not only more highly induced in WT compared to *Adrb*2^-/-^ cells at day 4 post-infection, it was also selectively included in genes constituent to WT path G, but not in *Adrb*2^-/-^ path E (S6 Table). Thus, the ADRB2 is involved in regulating the proper timing of gene expression patterns during early T cell priming, and the factors that correlated with these differences are known regulators of effector T cell development.

## Discussion

The sympathetic nervous system controls a broad range of behavioral and physiological processes. Given the significant innervation of secondary lymphoid tissues by sympathetic neurons, it is not surprising that this neurotransmitter pathway regulates immune function. Indeed, prior reports have shown a clear role for NE and the ADRB2 in suppressing a variety of acute immune functions [21], including acute cytokine secretion and lytic activity in CD8^+^ T cells [5–8]. In prior studies, the absence of adrenergic signaling *in vivo*, by chemical sympathectomy, increased IFN-γ producing CD8^+^ T cells after influenza infection [5]. Consistent with these findings, we recently demonstrated that antigen-induced cytokine secretion from CD8^+^ T cells was markedly decreased when mice were treated with a long-acting β2-agonist during VSV infection [6]. Although NE and ADRB2 agonists suppressed acute CD8+ T cell effector function in response to antigen receptor activation, it was not clear how adrenergic signaling influenced downstream effector T cell development. Our previous studies found that *in vitro* priming of naive CD8^+^ T cells into effector cells was not affected by NE during the differentiation stage [6]. In the current study, we uncovered a statistically significant role for ADRB2 signaling that regulated various aspects of CD8^+^ T effector cell responses to *in vivo* virus infection. First, we found that adrenergic signaling regulated CD25 expression and IL-2 secretion, which correlated with a reduced proportion of *Adrb2*^*-/-*^ to WT Ag-specific cells towards the end of the expansion phase *in vivo*. Although IL-2 treatment increased CD25 expression on responding effector Adrb2-/- cells in vivo, it was not sufficient to restore their expansion to WT levels, suggesting that the ADRB2 regulates other aspects of their responses. As CD25 expression correlates with memory cell development [22–24], it is possible that the transition from effector to memory cell development can be impacted by ADRB2 signaling. Second, our current work demonstrated that intrinsic ADRB2 signaling on CD8^+^ T cells regulates a dynamic program of gene expression that correlates with both their expansion and their time-dependent development into effector cells.

Gene expression is regulated by networks of interactions, and these networks show context-dependent adaptation [25,26]. The dynamic changes in gene expression that occurred in CD8^+^ T cells during their expansion into effector cells was generally preserved in *Adrb2*^*-/-*^ cells, as the main components of those gene clusters evolved over time in both WT and *Adrb2*^*-/-*^ cells. However, by monitoring the dynamic changes in gene expression that occurred throughout the effector expansion phase with SMARTS [15], we identified clusters of genes that were significantly dysregulated in the absence of the *Adrb2*. Further analyses predicted unique transcription factors that could be responsible for the regulation of those gene clusters based on combinatorial gene expression [27]. The main branch of genes that were induced in WT cells at d4 post-infection (path G) shared most of its genes with those induced in *Adrb2*^*-/-*^ cells at a later time (d5, path E). While most of the predicted regulators of those genes were common to both paths, some were more highly induced at d4 in WT cells, such as *Pax5* and *Mafb*. The d4 path G in WT cells was comprised of a variety of genes involved in major T cell fate-determining pathways, and the delay in their induction observed in *Adrb2*^*-/-*^ cells may indicate a critical role for *Adrb2* signaling in the temporal response to infection. It is unlikely that ADRB2 signaling regulates a single factor that solely orchestrates these branch points. Nonetheless, we speculate that the *Adrb2* regulated those factors during T cell proliferation to provide a temporal program of gene expression corresponding to their timely response to the pathogen. These mechanisms could include signaling pathways, transcriptional changes, post-translational modifications, epigenetic alterations, and asymmetric inheritance of fate-determining factors during cell division [23,28,29].

Previous studies have demonstrated an immunosuppressive role for ADRB2 signaling, acting acutely to dampen the magnitude of cytokine expression and lytic activity in pre-committed effector cells [8,30,31]. Interestingly, deletion of *Adrb2* also impacts the diurnal recirculation of T cells through secondary lymphoid tissues [32,33], indicating a role for ADRB2 signaling in circadian regulation. Indeed, we identified several core circadian genes, such as *Per2* and *Fbxl3*, differentially regulated at various time points of infection, suggesting a direct role for regulating these clock genes intrinsically in CD8^+^ T cells. The detailed temporal map of gene expression described here has identified a role for the ADRB2 in the primary response of acute CD8^+^ T effector cells to viral infection. Future studies will utilize these networks to identify how these pathways functionally regulate effector and memory cell development through the course of infection.

## Materials and methods

### Animals

BALB/cJ, Clone4-Tg (Cl4) [34], and *Adrb2*^-/-^ [35] mice were housed in specific pathogen-free conditions at the University of Texas Southwestern Medical Center Animal Research Center facilities. Adrb2^-/-^ mice bred onto the BALB/c background [36] were a kind gift from Dr. Virginia Sanders (Ohio State University), and Cl4 mice were purchased from Jax mice (Jackson laboratory). All experiments involving mice in this study were approved by the Institutional Animal Care and Use Committee of the University of Texas Southwestern Medical Center.

### Adoptive T cell transfer and VSV-HA infection

Recombinant VSV-HA expressing hemaglutinin from Influenza A PR/8 [10] was a kind gift from Dr. Elizabeth Ramsburg. CD8^+^ T cells were isolated from spleen and lymph nodes (axillary, brachial, inguinal, and superficial cervical) of WT and *Adrb2*^-/-^ Cl4 mice (7–12 weeks old) with a negative isolation kit according to the manufacturer’s instructions (Invitrogen #11417D). Donor T cells were derived from CD90.1 congenic animals in order to track their frequency in CD90.2 hosts. For co-transfer experiments, 2000 cells of a 1:1 mix of each genotype (1000 cells each) were intravenously (i.v.) injected into naive BALB/cJ mice in 100 μLs of sterile saline. One day later, each mouse was infected i.v. with 1e^6^ plaque-forming units (PFU) of VSV-HA. Mice were then sacrificed at the time points indicated post infection, and the frequency of transferred cells was monitored by flow cytometry by staining for CD90.1 and CD90.2.

For in vivo IL-2 activation, cohorts of mice were treated with either rat IgG2a/rmIL-2 (control) or with anti-IL-2/rmIL-2 for 3 consecutive days after infection with VSV-HA. Mice received mixtures of either 50 μg rat IgG2a + 1.5 μg rmIL-2 or 50 μg anti-IL-2 (clone S4B6) + 1.5 μg rmIL-2 [37].

### In vitro T cell assays

*In vitro* cytokine assays were performed as previously described [6]. Single cell suspensions from spleen and lymph nodes (axillary, brachial, inguinal and superficial cervical) were prepared separately and incubated at 2x10^6^/mL in the presence or absence of the CD8^+^ T cell-specific HA peptide (IYSTVASSL, 50 nM) for 21–24 hrs. For *in vitro* cell division assays, cells were pre-labeled with CFSE prior to activation and allowed to divide in culture for 72 hrs. Cells were stained for CD90.1 and CD25 and analyzed by FACS.

### RNASeq analysis

WT and *Adrb2*^-/-^ C4-Tg T cells were co-transferred to BALB/cJ recipients followed by infection with VSV-HA, as described above. RNA was isolated from cells prior to transfer and from CD90.1/2 FACS-purified cells isolated from cohorts of infected animals at incremental days post-infection. WT and *Adrb2*^-/-^ cells were distinguished based on single or co-expression of CD90.1 and CD90.2. Barcoded libraries were prepared from purified mRNA (New England Biolabs, #E7530S, #E7490S, #E7335S, and Axygen #MAG-PCR-CL-5) and sequenced on an Illumina HISEQ 2500.

Quality assessment of the RNASeq data was performed using NGS-QC-Toolkit [38]. Reads with more than 30% of nucleotides with Phred quality scores less than 20 were removed from further analysis. Quality filtered reads were then aligned to the mouse reference genome GRCm38 (mm10) using the HISAT (v 2.0.1) aligner [39] using default setting except for–library-type = fr-firststrand. Aligned reads were counted using featureCount (v1.4.6) [40] per gene ID. Differential gene expression analysis was performed using the R package edgeR [14] (v 3.8.6). For each comparison, genes were required to have 1 read in at least 1 sample to be considered as expressed. They were used for normalization factor calculation. Gene differential expression analysis was performed using GLM approach following edgeR analysis. Cutoff values of fold change greater than 2 and FDR less than 0.01 were then used to select for differentially expressed genes between sample group comparisons. Normalized gene FPKM values were averaged within groups for heatmap generation. MetaboAnalyst3.0 [41] was used to perform R-based principle component analysis (PCA) and integrated pathway analysis using the KEGG metabolic pathway database.

To perform the SMARTS analysis, we developed a new version of SMARTS, ’Supervised SMARTS’ that can use known class labels for building models. SMARTS uses an Input/Output Hidden Markov Model (IOHMM) approach to model the regulation of genes over time. Each SMARTS model reconstructs the regulatory activity of an ensemble of individual time series. SMARTS requires as input a mapping between transcriptional regulators and their gene targets. We generated such a mapping using the regulatory behavior identified in Best et al. in their S12 Table [16]. We used SMARTS to build two models, one from the three WT cells time series and the other from the three *Adrb2*^-/-^ cells time series. The SMARTS analysis covered all 5 time points, from 0–12 days post infection. We further used SMARTS to identify putative differentially active transcription factors between the two models. In brief, we identify transcription factors whose regulated genes can only have their gene expression patterns explained by the proper model. This criterion is evaluated using a permutation test to determine statistical significance. See Wise and Bar-Joseph, 2013 section 2.4 for complete details [15].

### Statistical analyses

Three different statistical tests were performed using the GraphPad Prism software. For simple pairwise comparisons, a Student’s two-tailed t-test was used. Otherwise, a one-way or two-way ANOVA was used followed by a Bonferroni posttest for pairwise comparisons within the groups, as indicated in the figure legends. Differences were considered significant at p ≤ 0.05.

## Supporting information

S1 Table Table of all differentially expressed genes between WT and *Adrb2*^-/-^ cells at all timepoints. (XLSX)

S2 Table KEGG analysis of differentially expressed genes between WT and *Adrb2*^-/-^ cells at each timepoint. (XLSX)

S3 Table Tables of genes in each path of the *Adrb2*^+/+^ SMARTS model. (XLSX)

S4 Table Tables of genes in each path of the *Adrb2*^-/-^ SMARTS model. (XLSX)

S5 Table Tables of transcription factor predictions for each path of the *Adrb2*^+/+^ SMARTS model. (XLSX)

S6 Table Tables of transcription factor predictions for each path of the *Adrb2*^-/-^ SMARTS model. (XLSX)

S7 Table Table of transcriptional regulator (TR) prediction differences between *Adrb2*^+/+^ and *Adrb2*^-/-^ models. (XLSX)

S8 Table Table of genes included in unique and shared paths by Venn analysis. (XLSX)
